# Transdermal diclofenac versus oral paracetamol-ibuprofen combination for postoperative pain following surgical extraction of mandibular third molar: a randomized controlled trial

**DOI:** 10.1097/MS9.0000000000004076

**Published:** 2025-10-14

**Authors:** Niroj Khanal, Mehul Rajesh Jaisani, Ashok Dongol, Pradeep Acharya, Anjani Kumar Yadav, Asish Subedi, Bibek Kattel, Siddhartha Rai

**Affiliations:** a Department of Oral and Maxillofacial Surgery, BP Koirala Institute of Health Sciences, Dharan, Nepal; b Department of Anesthesiology and Critical Care, BP Koirala Institute of Health Sciences, Dharan, Nepal; c College of Dental surgery, BP Koirala Institute of Health Sciences, Dharan, Nepal

**Keywords:** ibuprofen, impacted teeth, mandible, paracetamol, postoperative pain, transdermal diclofenac patch

## Abstract

**Background:**

Postoperative pain management following surgical extraction of mandibular third molars require effective analgesic options with favorable safety profiles. This study compares the analgesic efficacy and side effect profiles of a transdermal diclofenac patch versus oral combination of paracetamol and ibuprofen.

**Materials and methods:**

Total of 108 patients indicated for surgical extraction of mandibular third molars were randomized into two groups: Group-A (*n* = 54) received an oral combination of paracetamol (500 mg) and ibuprofen (400 mg), and Group-B (*n* = 54) received a diclofenac transdermal patch (200 mg). Pain [numeric rating scale (NRS)], rescue analgesics, and side effects were assessed at 12, 24, and 72 hours postoperatively. Data were analyzed using descriptive statistics and inferential analysis, including independent *t*-test, analysis of variance, and chi-square test, with a significance threshold at *P* < 0.05.

**Results:**

Both groups showed significant pain scores reduction over time. Mean NRS scores at 12, 24 and 72 hours were 3.06, 1.15, and 0.69 (Group-A) vs. 3.46, 1.22, and 0.67 (Group-B) respectively with no statistically significant differences in pain scores between two groups at any time. However, the incidence of side effects, particularly gastric irritation, was significantly higher in Group-A compared to Group-B.

**Conclusion:**

Both the transdermal diclofenac patch and the oral combination of paracetamol and ibuprofen are effective in managing postoperative pain following mandibular third molar extraction. However, the transdermal diclofenac patch has significantly fewer gastrointestinal side effects, making it a preferable option for patients at risk of gastric irritation.

## Introduction

The International Association for the Study of Pain (IASP) 2020 redefined pain as “An unpleasant sensory and emotional experience associated with, or resembling that associated with, actual or potential tissue damage”^[1]^. Pain is commonly referred to as a defense mechanism, triggered by environmental changes following an injury to responsive tissue. This type of pain increases the responsiveness of central and peripheral pain pathways by modulating the somatosensory system^[2]^. Pain is one of the most commonly experienced symptoms after oral and maxillofacial surgical procedures, making it a major concern for the surgeons. Control or elimination of pain is crucial, as it is a common postoperative complication following the surgical removal of the mandibular third molar. If the pain is not relieved or is recurring, it may cause physical discomfort and psychological distress, decreasing the quality of treatment and increasing morbidity and mortality^[2,3]^.

**HIGHLIGHTS**

- The transdermal delivery system avoids first-pass metabolism, maintains steady drug levels, and reduces systemic side effects, offering a practical alternative to oral nonsteroidal anti-inflammatory drugs (NSAIDs) for postoperative pain management.
- Both transdermal diclofenac and oral paracetamol-ibuprofen provided similar pain relief after third molar surgery, with no significant difference in pain scores at 12, 24, or 72 hours postoperatively.
- The transdermal diclofenac patch caused significantly fewer gastrointestinal side effects compared to the oral NSAID combination, making it safer for patients prone to gastric irritation.
- Although slightly more patients using the diclofenac patch needed rescue pain medication, the difference was not statistically significant, confirming both treatments were similarly effective.

Nonsteroidal anti-inflammatory drugs (NSAIDs) are the most commonly prescribed for postoperative pain management after third molar surgery due to their effective antipyretic, anti-inflammatory, and analgesic properties. Commonly prescribed NSAIDs include oral ibuprofen combined with paracetamol and oral diclofenac sodium. NSAIDs effectively relieve mild-to-moderate postoperative pain^[3]^. They inhibit either Cyclo-oxygenase-1 (COX-1), Cyclo-oxygenase-2 (COX-2), or both, which are responsible for the synthesis of prostaglandins (PGs) evident in different pathological contexts, with COX-2 showing heightened expression in these situations. NSAIDs can be administered through oral, parenteral, inhalation, and transdermal routes, with oral administration being the most common. However, the oral route carries the risk of first-pass metabolism leading to significant drug loss before systemic absorption. The overuse of oral NSAIDs (Diclofenac) can cause adverse cardiac, renal, and gastrointestinal effects. Parenteral drug delivery can cause significant discomfort, and a rapid increase in plasma drug concentration can lead to adverse effects^[4]^.

The transdermal delivery system has gained popularity over the last decade as it can overcome the pharmacokinetic barriers of oral and parenteral routes. It bypasses the first-pass metabolism, allows for gradual and controlled drug absorption, maintains constant plasma concentration for an extended duration, reduces patient dependence on drug doses, and avoids gastric discomfort. Further, it provides the flexibility of discontinuing the drug administration by removing the patch from the skin^[5]^. Topically applied NSAIDs result in plasma concentration that is less than 5% of those achieved with oral administration^[6]^. Lower plasma drug concentrations limit systemic side effects and have the added benefit of enhanced localized action at the inflammation site. Thus, transdermal delivery is associated with fewer gastrointestinal adverse effects and is particularly useful in patients intolerant to the oral route^[7]^.

The study aims to compare the analgesic effectiveness of a diclofenac transdermal patch (200 mg) and an oral combination of paracetamol (500 mg) and ibuprofen (400 mg) following the surgical removal of the mandibular third molar.

## Materials and methods

**Figure 1.**
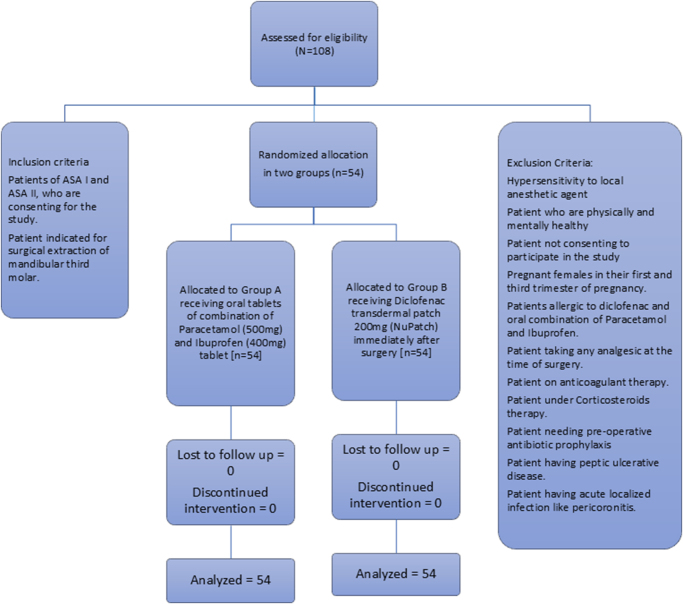
Consolidated standards of reporting trials (CONSORT) flow chart. N, number.

The prospective comparative study was approved by the Institutional Review Committee (IRC) of B.P. Koirala Institute of Health Sciences, Dharan (Ref no. IRC/2223/022) and registered in ClinicalTrails.gov (identifier: NCT06146491). The randomized clinical trial was conducted from 1 September 2022 to 31 August 2023 in patients indicated for surgical extraction of mandibular third molar at the Department of Oral and Maxillofacial Surgery, College of Dental Surgery, BP Koirala Institute of Health Sciences, Dharan, Nepal. The trial was conducted under the supervision of one professor, two additional professors, and one assistant professor from the Department of Oral and Maxillofacial Surgery, along with one additional professor from the Department of Anesthesiology. All surgical procedures were performed by a resident in Oral and Maxillofacial Surgery under direct oversight to ensure adherence to standardized protocols and consistency in surgical technique. Due to paucity of prior research, the expected sample size was determined from hospital records rather than formal power calculation. Records indicated an average of 12 impacted mandibular third-molar surgeries were performed per month, giving a maximum feasible sample size of 108 cases over 9 months. Patients of the American Society of Anesthesiologists (ASA) I and ASA II, indicated for surgical extraction of the mandibular third molar and consenting to participate, were enrolled consecutively. Computer-based randomization was done, and patients were assigned to two groups. A total of 108 patients were enrolled, with 54 assigned per group without dropouts. Individuals received one of the other interventions according to their group allocation (Fig. 1).

The work has been reported in line with Consolidated Standards of Reporting Trials (CONSORT) Guidelines^[8]^ and in compliance with the Transparency in the reporting of Artificial Intelligence (TITAN) Guidelines 2025 for use of artificial intelligence in scientific writing^[9]^.

## Interventional procedure

After explaining the study protocol, verbal and written consent were obtained from the patient. Patients with a known history of an allergy or hypersensitivity to lignocaine, bleeding disorder, patients under corticosteroids, pericoronitis, and pregnant and lactating mothers were excluded.

The surgical procedure was performed by the same surgeon under the same environment using a standard aseptic technique. Local anesthesia was administered using regional blockade of the inferior and lingual alveolar nerves, with supplementary buccal nerve infiltration using 2% lidocaine and 1:200 000 epinephrine. The standard surgical procedure for the surgical removal of impacted third molars was used for both groups. After achieving optimum anesthesia, the incision was given as per Ward’s incision technique. Howarth’s Periosteal Elevator was used to reflect a full-thickness mucoperiosteal flap and was retracted with Austin’s retractor. Buccal and distal bone removal was done with a round bur (No. 04) on a straight handpiece under constant irrigation with 0.9% sterile normal saline solution, and guttering was done a little beyond the bifurcation. After the tooth extraction socket was inspected, irrigated, and flap sutured with a 3-0 vicryl (Reverse Cutting). One suture was placed just distal to the lower second molar, and another on the distal aspect of the extraction socket.

**Figure 2.**
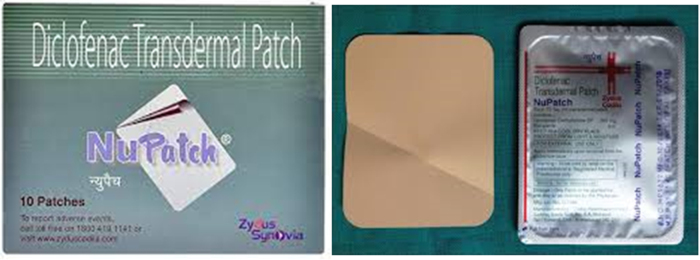
Transdermal diclofenac patch.

**Figure 3.**
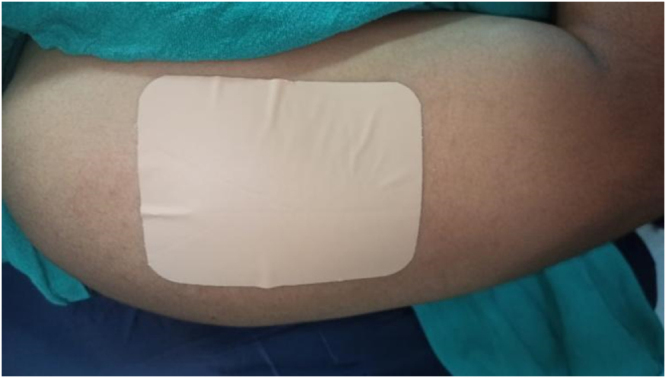
Site of application of patch on right shoulder.

The duration of the surgical procedure was recorded from the time of incision to the last suture. After the surgical procedure, Group A was prescribed a combination of Paracetamol (500 mg) and Ibuprofen (400 mg) tablets orally 30 minutes after the procedure and followed by a single tablet every 8 hours for 3 days, while Group B was prescribed a Diclofenac transdermal patch 200 mg (NuPatch) (Fig. 2) immediately after surgery. A diclofenac transdermal patch was placed on the right arm (Fig. 3) and was replaced after 24 hours for three consecutive days. The patients and their caregivers were instructed and demonstrated how to apply and remove the diclofenac transdermal patch at the time of the first application. Patients were advised to rinse with 0.12% Chlorhexidine digluconate aqueous solution to control dental plaque from the second day, every 12 hours for 7 days. Amoxicillin trihydrate 500 mg 8 hourly or Azithromycin 500 mg 24 hourly for those who were allergic to Penicillin, Metronidazole 400 mg 8 hourly was prescribed.

Pain was evaluated using a 10-point numeric rating scale (NRS), where 0 indicated no pain and 10 indicated the worst possible pain. At specified time intervals after the application of the transdermal patch, patients reported their pain scores over a telephonic interview conducted by a separate evaluator, a dental officer.

Tramadol 50 mg capsule as a rescue analgesic was given if the pain score was greater than or equal to seven. Patients were instructed to report in the event of any complications, such as uncontrolled bleeding and unbearable pain. The pain was assessed at three time intervals: 12, 24, and 72 hours post-operative. The number of rescue analgesics consumed by the patients was also recorded. Any adverse effects like allergic reaction to the drug used, nausea, vomiting, gastric irritation, etc., were also recorded.

## Blinding and confounders

Single blinding was adopted to minimize bias. The evaluator (a dental officer) who assessed post-operative pain scores via telephonic interview was blinded to the group allocation. The surgeon performing the procedures and the patients themselves were not blinded due to the obvious difference between oral and transdermal drug administration.

All procedures were performed by a single experienced surgeon using standardized aseptic techniques and anesthesia protocols to minimize confounding. Randomization ensured balanced distribution of demographic and clinical variables across groups. Post-operative care, including antibiotics and a chlorhexidine rinse, was standardized for all participants. Evaluator-related bias was minimized through single blinding and prior training of the assessor.

## Data management and statistical analysis

All data were collected and managed by the principal investigator, with monthly checks to ensure accuracy. Data were entered into Microsoft Excel and Statistical Package for Social Sciences (SPSS) version 26 was used for analysis. Categorical variables were coded for ease of data entry and analysis. Supervision and monitoring of the data entry process were performed regularly by the guide and co-guide. Descriptive statistics, including mean, standard deviation, range, and percentages, were calculated to summarize the data. For inferential analysis, the independent t-test was used to analyze the type of impaction and compare mean [standard deviation (SD)] post-operative outcomes, and compare significant differences in the use of rescue analgesics between groups. Additionally, the independent t-test was used to compare the mean pain scores between Group A and Group B at each post-operative time point. Lastly, a chi-square test was applied to compare the occurrence of adverse effects between the groups.

## Observations and results

A total of 108 patients were enrolled in the study. After randomization, each group has 54 patients.

## Demography

**Figure 4.**
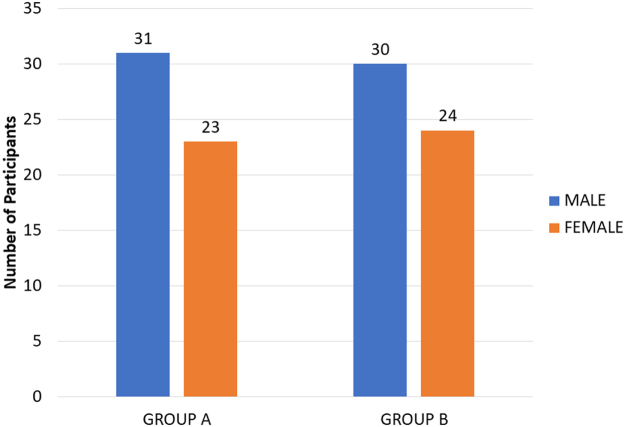
Bar diagram showing gender distribution.

The study included 61 men and 47 women with an average age of 28.34 ± 9.04 years, ranging from 19 to 69 years. Group A had 31 (57.4%) males and 23 (42.6%) females, whereas Group B had 30 (55.5%) males and 24 (44.5 %) females, with no statistically significant differences between the groups (Fig. 4).

Mesioangular impaction was the most common type of impaction in both groups (Table 1). The types of impactions were evaluated using an independent t-test. There was a statistically significant difference in the distoangular type of impaction between the two groups (*P*-value = 0.004), with Group B having more cases of distoangular impaction than Group A (Table 1).

**Table 1:** Distribution of types of tooth impaction in Group A and Group B

Type of impaction	Group A (*n* = 54)	Group B (*n* = 54)	*P*-value*
Mesioangular	30 (55.6%)	20 (37.0%)	0.054
Distoangular	5 (9.3%)	17 (31.5%)	0.004
Vertical	11 (20.4%)	7 (13.0%)	0.302
Horizontal	8 (14.7%)	10 (18.5%)	0.606

^*^ Independent *t*-test.

*P*-value < 0.05 is statistically significant.

## Operative time (in minutes) in Group A and Group B

In both groups, distoangular impaction took the longest (B: 35 minutes and A: 33.20 minutes), followed by horizontal impaction (A: 26.25 minutes and B: 30.5 minutes). The overall mean (SD) operative time (Table 3) for groups A and B was 24.2 minutes (7.0) and 29.4 minutes (7.5), respectively. A two-way analysis of variance (ANOVA) revealed a significant difference in operative time between the two groups for different types of impactions (*P* = 0.022) (Table 2). The mean (SD) of the overall operative time for group A is compared in Table 3.

**Table 2:** Comparison mean operative time (in minutes) in Groups A and B

Type of impaction	Group	Mean (minutes)	Standard deviation (minutes)
Mesioangular	A (*n* = 30)	22.33	5.68
	B (*n* = 20)	22.50	4.73
Distoangular	A (*n* = 5)	33.20	11.77
	B (*n* = 17)	35.00	5.86
Vertical	A (*n* = 11)	22.33	5.68
	B (*n* = 7)	22.50	4.73
Horizontal	A (*n* = 8)	26.25	3.53
	B (*n* = 10)	30.50	5.98

**Table 3:** Comparison of overall operative time in Group A and Group B

Overall operative time (in minutes)	Group A	Group B	*P*- value
Mean (SD)	24.28 (7.08)	29.26 (7.54)	<0.001

Independent *t*-test.

*P*-value < 0.05 is statistically significant.

## Pain scores at different time points for Groups A and B

Pain scores at 12, 24, and 72 hours were evaluated (Table 4). The difference in NRS pain scores at different intervals between the two groups was statistically not significant.

**Table 4:** Comparison of mean (SD) post-operative pain score after 12, 24, and 72 hours in Group A and Group B

Mean (SD)	Group A [*n* = 54]	Group B [*n*= 54]	*P*-value
Pain after 12 hours	3.06 (1.59)	3.46 (2.16)	0.267
Pain after 24 hours	1.15 (0.68)	1.22 (0.98)	0.651
Pain after 72 hours	0.69 (0.57)	0.67 (0.67)	0.726

Independent *t*-test.

*P*-value < 0.05 is statistically significant

**Figure 5.**
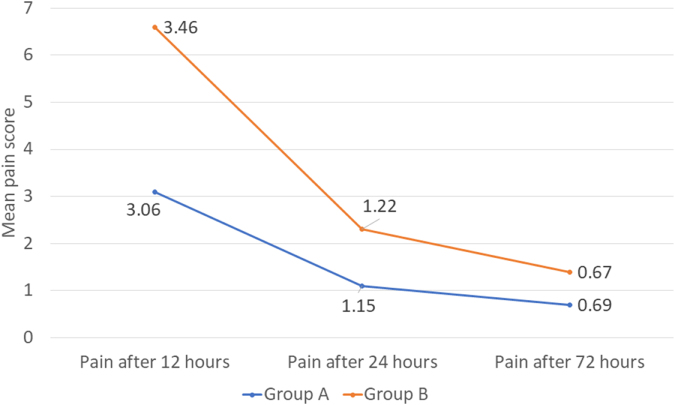
Line graph showing comparison of mean post-operative pain score.

One-way repeated measure of ANOVA was used to compare patients’ mean pain scores in three different post-operative periods in both groups (Table 5). In Group A, the ANOVA results showed that there was a significant mean difference between all the post-operative periods’ df (1.23, 65.51), *P* < 0.0001. In Group B, the ANOVA results showed a significant mean difference between all the post-operative periods’ df (1.26, 67.27), *P* < 0.0001. In both groups, the pairwise comparison revealed that there was a significant mean difference between each of the post-operative periods, i.e., there was a significant difference in mean scores between 12, 24, and 72 hours post-operatively (Fig. 5).

**Table 5:** Comparison of mean pain score at 12, 24, and 72 hours in Groups A and B

Group	Post-operative time	Mean pain score	Standard deviation	Significance
A	Pain after 12 hours	3.06	1.59	#&
	Pain after 24 hours	1.15	0.68	*&
	Pain after 72 hours	0.69	0.57	*#
B	Pain after 12 hours	3.46	2.16	#&
	Pain after 24 hours	1.22	0.98	*&
	Pain after 72 hours	0.67	0.67	*#

ANOVA.

*P* value <0.05.

* *P* value significant with 12 hours post-operative.

# *P* value significant with 24 hours post-operative.

& *P* value significant with 72 hours post-operative.

## Side effects seen in Group A and Group B

Statistical analysis using the chi-square test revealed that the difference in adverse effects between the oral combination of Paracetamol and Ibuprofen (Group A) and the Diclofenac transdermal patch (Group B) was statistically significant (*P*-value < 0.001) (Table 6).

**Table 6:** Comparison of side effect seen in Group A and Group B

Side effect [*n* (%)]	Group A (*n* = 54)	Group B (*n* = 54)	*P*-value	Chi square
Present	31 (57.5%)	5 (9.2%)	<0.001	28.17
Absent	23 (42.5%)	49 (90.8%)		

Chi-square test.

*P*-value < 0.05 is statistically significant.

## Rescue analgesics

The total number of rescue analgesics taken was higher in Group B (six instances) compared to Group A (four instances). The 95% confidence interval for this difference was 0.2 to 2.4. Statistical analysis using the chi-square indicated that the difference in the use of rescue analgesics (Tramadol 50 mg) between patients using the oral combination of Paracetamol and Ibuprofen and those using the Diclofenac transdermal patch was not statistically significant (*P* = 0.507) (Table 7).

**Table 7:** Comparison of number of rescue analgesics taken in Group A and Group B

Tramadol [*n* (%)]	Group A (*n* = 54)	Group B (*n* = 54)	*P*-value	Chi square
Required	4 (8%)	6 (11%)	0.507	0.44
Not required	50 (92%)	48 (89%)		

Chi-square test.

*P*-value < 0.05 is statistically significant.

## Discussion

The surgical extraction of third molars is among the most commonly performed oral surgeries globally,^[10]^ often indicated for conditions like dental caries, periodontal problems, pericoronitis, cyst formation, and orthodontic purposes. Despite precise surgical techniques, patients usually experience notable postoperative discomfort, including pain, swelling, and trismus, largely attributable to the region’s loose connective tissue and abundant vascularity^[11]^. The inflammatory process initiated by surgical trauma triggers post-operative discomfort, particularly pain. These post-operative complications can cause significant distress, and any compromises on the quality of life, even for a few days, are often unacceptable.

Effective management of post-operative pain is challenging and is continuously evolving^[12]^. NSAIDs are considered ideal analgesic agents for controlling pain after surgical removal of mandibular third-molar impaction^[13,14]^. As they reduce both pain and inflammation.

NSAIDs can be administered via oral, parenteral, inhalation, and transdermal routes. While the oral route is the most common method of drug administration, transdermal delivery offers several distinct advantages. It provides non-invasive and painless drug delivery, ensuring controlled release over an extended period with the added benefit of flexibility to terminate administration abruptly if needed. Compared to oral medications or supplements, it has fewer side effects, improving patient compliance due to its ease of use. Additionally, transdermal systems prolong the activity of drugs with short plasma half-lives through controlled release from the therapeutic delivery system. This method is particularly suitable for patients with cognitive impairments or disabilities who may face challenges in self-medicating^[5,15–17]^.

Although controversial in acute pain treatment, topical NSAIDs are still widely prescribed in some parts of the globe to test the efficacy of analgesics for acute dental pain^[18]^. It is well documented that pain following surgical removal of the third molar is of short duration and reaches its maximum in the early postoperative period^[7,19,20]^.

Analgesic patches are well-established approaches to topical NSAID administration. The transdermal patch is used to relieve mild to moderate pain, applied once daily for 24 hours, and provides rapid pain relief with minimal or no side effects. It comes in two strengths: 100 and 200 mg. The 100-mg patch is 50 cm^2^ and the 200-mg patch is 75 cm^2^. The patch achieves plasma levels ranging from 20 to 50 ng/mL, which is lower compared to the oral route, but these levels are sustained for a longer period^[5]^.

In this study, selection bias has been controlled by randomization of the participants. The total number of participants was 108, which was greater than most of the reported studies^[2,20–22]^. For comparison, Dastagir *et al*^[23]^ included 100 participants, Jitender *et al*^[24]^ included 80 participants, Aimuamwosa *et al*^[5]^ included 68 participants, and Bachalli *et al*^[3]^ included 20 participants. The majority of the patients were male. However, there is no uniform data regarding gender distribution in similar study designs^[3,5,25,26]^. Further, the statistically insignificant gender distribution suggests that gender did not influence the results of the study. The age group in the study ranges from 19 to 59 years old. We selected the case above 18 years because the eruption of mandibular third molars is often complete around the age of 20 years^[27]^. We observed that the age of the patients did not influence the results of the study.

This study includes all variants of impaction in variable proportions. In contrast, most studies included only mesioangular impaction^[1,3,5,24]^. Therefore, our sample represents the general population more accurately. Besides distoangular impaction, all other variants of impaction were equally distributed between the two groups. There was a significant difference in overall operating time between the groups. This difference could be attributed to the greater number of distoangular cases in the transdermal diclofenac group. Evidences suggest distoangular impactions are the most difficult to extract, requiring more time^[27]^.

The evaluation of pain is always subjective, but can be assessed using various scales such as the visual analog scale, verbal rating scale, pain intensity scale, pain relief scale, and NRS. The NRS is an 11-point scale ranging from 0, referring to no pain, and 10, referring to the worst pain. The NRS has shown high correlations with other pain assessment tools in several studies^[28]^. Its feasibility and good compliance have also been proven^[29]^ as it can be recorded over telephone interviews. Therefore, we preferred the NRS Scale for pain evaluation^[30]^. The 12-hour post-operative mean NRS scores were higher in both groups compared to the mean pain score at 24 and 72 hours. The higher pain score at 12 hours is due to trauma-induced inflammation following impacted mandibular third molar removal^[31]^. Despite the peak pain score at 12 hours, both the oral combination of paracetamol and ibuprofen and the transdermal diclofenac patch were equally effective in controlling pain with no statistically significant difference between the two groups. This observation is consistent with findings from other studies^[2,3,5]^. We observed a gradual decrease in pain scores at 24 and 72 hours in both groups, with both analgesics remaining equally effective in controlling pain, as there was no statistically significant difference between the groups. The decrease in pain score can be attributed to the gradual reduction in inflammation over time, as also reported by other investigators^[2,23,24]^. Despite significantly higher operating time in the transdermal diclofenac group, leading to more inflammation, we observed no significant difference in pain reported by patients between the two groups. This suggests that the diclofenac patch is potent enough to control post-operative pain secondary to intense inflammation.

In this study, a significant number of patients reported gastrointestinal irritation following the use of an oral combination of paracetamol and ibuprofen compared to patients with a transdermal diclofenac patch, which could be related to the inhibition of COX-1-mediated synthesis of gastro-protective PGs, with local action inducing back diffusion of H^+^ ions in the gastric mucosa also playing a role. A deficiency of PGs reduces mucus and HCO_3_− secretion and tends to enhance acid secretion^[4]^. Similar observations have been reported by other investigators^[2,3,5,21–26,32]^.

In this study, rescue analgesics were taken more frequently in the transdermal group; however, this was not statistically significant. This difference may be due to the greater number of distoangular impactions and the increased operative time of transdermal diclofenac. To the best of our knowledge, no studies have compared two different analgesics administered through different routes; however, many previous studies have been reported with similar study designs using diclofenac through two different routes^[2,3,5,12,21,22,24,26]^. These studies observed that a greater number of supportive medications were required in the diclofenac patch group than in the oral diclofenac group.

Though the diclofenac patches were more expensive than ibuprofen and paracetamol combination tablets. They are an effective alternative to ibuprofen and paracetamol tablets, controlling pain with significantly lower adverse reactions.

## Strengths, limitations, and future recommendations

This study has several notable strengths. Unlike previous research, it included all variants of mandibular impaction, thereby providing a more realistic representation of the target population. All surgical extractions were performed by the same surgeon under similar environmental conditions, minimizing operator- and setting-related bias. Postoperative pain scores were recorded by an independent evaluator, further reducing assessment bias. In addition, randomization ensured that baseline variables between the two groups were comparable. However, the study also has certain limitations. The comparison involved two different drugs delivered via two different routes, and these were administered at different postoperative time intervals, which may have influenced the outcomes. In addition, the distoangular impactions were unevenly distributed between the groups, which may have influenced the results. Furthermore, the transdermal diclofenac patch is relatively expensive and less easily available than the oral combination of ibuprofen and paracetamol, potentially limiting its widespread clinical use. Based on these findings, we recommend that future studies should explore other analgesics with different routes of administration. Moreover, transdermal diclofenac patches can be considered as an effective alternative to the oral combination of ibuprofen and paracetamol for postoperative pain management following the surgical extraction of mandibular third molars.

## Summary and conclusion

Transdermal diclofenac patches offer a promising alternative to oral NSAIDs for post-extraction pain management, providing comparable efficacy with enhanced patient comfort and fewer systemic side effects. Their sustained release and non-invasive administration address key limitations of traditional analgesics, though cost and availability may hinder widespread adoption. Future studies with larger, diverse populations are needed to confirm these findings and explore broader applications. Clinicians should consider transdermal diclofenac as a viable option for patients seeking needle-free, convenient pain relief after third molar surgery.

## Data Availability

All the relevant data have been included in the manuscript itself.

## Research registration unique identifying number (UIN)

(1) Name of the registry: Clinicaltrials.gov and IRC. (2) Unique Identifying number or registration ID: identifier: NCT06146491 IRC: Ref no IRC/2223/022. (3) Hyperlink to your specific registration (must be publicly accessible and will be checked): https://inclinicaltrials.com/pain-postoperative/NCT06146491/.

## References

[1] International Association for the Study of Pain (IASP). IASP’s proposed new definition of pain released for comment. Washington, D.C.: IASP; 2019.

[2] Deepika S Sathiyapriya S Nimmy P. Comparison of diclofenac transdermal patch against oral diclofenac for pain control following removal of mandibular impacted third molars: a systematic review. Int J Recent Adv Multidiscip Top 2022;3:27–30.

[3] Bachalli PS Nandakumar H Srinath N. A comparative study of diclofenac transdermal patch against oral diclofenac for pain control following removal of mandibular impacted third molars. J Maxillofac Oral Surg 2009;8:167–72. 23139499 10.1007/s12663-009-0041-8 PMC3453945

[4] Tripathi KD. Essentials of Medical Pharmacology. 7th ed. Jaypee Brothers Medical Publishers, New Delhi; 1985:193–209.

[5] Aimuamwosa OD Ekaniyere EB Obuekwe ON. Transdermal patch and oral routes of administration of diclofenac for the control of postoperative sequelae after third molar surgery: a single-blinded, randomized clinical trial. J Dent Oral Disord 2020;6:1139.

[6] Hupp JR Tucker MR Ellis E. Contemporary Oral and Maxillofacial Surgery. 7th ed. Elsevier, St. Louis; 2019:186–88.

[7] Zuniga JR Phillips CL Shugars D. Analgesic safety and efficacy of diclofenac sodium softgels on postoperative third molar extraction pain. J Oral Maxillofac Surg 2004;62:806–15. 15218558 10.1016/j.joms.2003.12.019

[8] Cheng A Kessler D Mackinnon R. Reporting guidelines for health care simulation research: extensions to the CONSORT and STROBE statements. Adv Simul 2016;1:1–3. 10.1186/s41077-016-0025-y PMC5806464 29449994

[9] Agha RA Mathew G Rashid R. Transparency in the reporting of artificial intelligence–the TITAN guideline. Prem J Sci 2025;10:100082.

[10] Enabulele JE Obuekwe ON. Prevalence of caries and cervical resorption on adjacent second molars associated with impacted third molars. J Oral Maxillofac Surg Med Pathol 2017;29:301–05.

[11] Sisk AL Hammer WB Shelton DW. Complications following removal of impacted third molars: the role of the experience of the surgeon. J Oral Maxillofac Surg 1986;44:855–59. 3464711 10.1016/0278-2391(86)90221-1

[12] Swaminathan C Comparative evaluation of transdermal diclofenac and oral diclofenac in management of postoperative pain in bilateral extractions: an *in vivo* study [dissertation]. Kulasekharam: Sree Mookambika Institute of Dental Sciences; [year not provided].

[13] Joshi A Parara E Macfarlane TV. A double-blind randomized controlled clinical trial of the effect of preoperative ibuprofen, diclofenac, paracetamol with codeine, and placebo tablets for relief of postoperative pain after removal of impacted third molars. Br J Oral Maxillofac Surg 2004;42:299–300. 15225946 10.1016/j.bjoms.2004.02.004

[14] Moore RA Tramer MR Carroll D. Quantitative systematic review of topically applied non-steroidal anti-inflammatory drugs. Bmj 1998;316:333–38. 9487165 10.1136/bmj.316.7128.333 PMC2665568

[15] Robinson JR Lee HL. Controlled Drug Delivery: Fundamentals and Applications. 2nd ed. Marcel Dekker, New York; 2007:524–52.

[16] Aqil M Sultana Y Ali A. Matrix-type transdermal drug delivery systems of metoprolol tartrate: *in vitro* characterization. Acta Pharm 2003;53:119–25. 14764246

[17] Kumar V Gupta S Verma R. Evaluation of the role of transdermal diclofenac patch (Nupatch) in management of pain in postoperative patients. Int J Contemp Med Res 2017;4:493–96.

[18] Pozos-Guillen A Martinez-Rider R Aguirre-Banuelos P. Pre-emptive analgesic effect of tramadol after mandibular third molar extraction: a pilot study. J Oral Maxillofac Surg 2007;65:1315–20. 17577495 10.1016/j.joms.2006.10.079

[19] Yamashita Y Sano N Shimohira D. A parallel-group comparison study of celecoxib with loxoprofen sodium in third mandibular molar extraction patients. Int J Oral Maxillofac Surg 2014;43:1509–13. 25270186 10.1016/j.ijom.2014.09.002

[20] Meechan JG Seymour RA. The use of third molar surgery in clinical pharmacology. Br J Oral Maxillofac Surg 1993;31:360–65. 8286289 10.1016/0266-4356(93)90191-x

[21] Selvi UP Kamatchi D Babu C. Comparison of transdermal diclofenac patch with intramuscular diclofenac injection as an analgesic modality following surgical extraction of impacted mandibular third molars: a crossover efficacy trail. Int J Sci Study 2016;4:117–23.

[22] Bhaskar H Kapoor P Ragini. Comparison of transdermal diclofenac patch with oral diclofenac as an analgesic modality following multiple premolar extractions in orthodontic patients: a crossover efficacy trial. Contemp Clin Dent 2010;1:158–63. 22114407 10.4103/0976-237X.72783 PMC3220102

[23] Dastagir F Balamurugan R. Comparing the analgesic safety and efficacy of diclofenac sodium tablet vs transdermal diclofenac on postoperative third molar extraction pain, swelling, and trismus. J Dentomaxillofac Sci 2019;4:67–70.

[24] Jitender J Arora V Singh R. Assessment of efficacy of diclofenac sodium in oral and transdermal patch in the management of postoperative pain following surgical removal of impacted mandibular third molars. Int J Health Sci 2022;6:9329–34.

[25] Raja Rajeswari S Gowda T Kumar T. Analgesic efficacy and safety of transdermal and oral diclofenac in postoperative pain management following dental implant placement. Gen Dent 2017;65:69–74. 28682286

[26] Diwan V Srinivasa ST Ramreddy YK. A comparative evaluation of transdermal patch with oral diclofenac sodium as an analgesic drug following periodontal surgery: a randomized controlled clinical trial. Indian J Dent Res 2019;30:57–60. 30900658 10.4103/ijdr.IJDR_84_17

[27] Peterson LJ. Peterson’s Principles of Oral and Maxillofacial Surgery. 3rd ed. Wolters Kluwer, Philadelphia; 2012:122.

[28] Jensen MP Karoly P Braver S. The measurement of clinical pain intensity: a comparison of six methods. Pain 1986;27:117–26. 3785962 10.1016/0304-3959(86)90228-9

[29] Closs SJ Barr B Briggs M. A comparison of five pain assessment scales for nursing home residents with varying degrees of cognitive impairment. J Pain Symptom Manage 2004;27:196–205. 15010098 10.1016/j.jpainsymman.2003.12.010

[30] Korff M Jensen MP Karoly P. Assessing global pain severity by self-report in clinical and health services research. Spine (Phila Pa 1976) 2000;25:3140–51. 11124730 10.1097/00007632-200012150-00009

[31] Weil K Hooper L Afzal Z. Paracetamol for pain relief after surgical removal of lower wisdom teeth. Cochrane Database Syst Rev 2007:CD004487. 17636762 10.1002/14651858.CD004487.pub2 PMC7388061

[32] Krishnan S. Transdermal diclofenac patch for control of post-extraction pain: a pilot randomized controlled double-blind study. J Maxillofac Oral Surg 2015;19:5–12. 10.1007/s10006-013-0422-5 23852077

